# Efficacy and safety of first-line treatments for recurrent or metastatic nasopharyngeal carcinoma: a systematic review and network meta-analysis

**DOI:** 10.3389/fimmu.2025.1485609

**Published:** 2025-06-09

**Authors:** Tongze Cai, Caiyue Lin, Qiongqian Li, Juanmei Mo, Jinghui Zheng, Jianlong Zhou

**Affiliations:** ^1^ Guangxi International Zhuang Medicine Hospital Affiliated to Guangxi University of Chinese Medicine, Nanning, China; ^2^ Graduate School, Guangxi University of Chinese Medicine, Nanning, China

**Keywords:** nasopharyngeal carcinoma, first-line, recurrent, metastatic, network meta-analysis

## Abstract

**Background:**

To compare the efficacy and safety of first-line treatments for recurrent or metastatic nasopharyngeal carcinoma (RM-NPC).

**Methods:**

We searched databases, including PubMed, Embase, Cochrane Library, ClinicalTrials.gov, and major international conferences, to identify comparative randomized controlled trials (RCTs) for the first-line treatment of patients with nasopharyngeal carcinoma who have metastasis or recurrence from inception to March 1, 2024. Then, we conducted a Bayesian network meta-analysis and systematic review of RCTs that met the specified inclusion criteria. By calculating the surface under the cumulative ranking curve (SUCRA) for each treatment, we determined their relative advantage: the higher the SUCRA score, the more likely that treatment is to be the optimal choice.

**Results:**

Seven RCTs were included, which involved 1495 patients who received 8 different treatment regimens. Overall, programmed cell death protein 1 (PD-1) inhibitors combined with chemotherapy could be the optimal treatment for patients with RM-NPC. Chemotherapy combined with radiotherapy has a tendency to improve progression-free survival and overall survival. The safety assessment showed no significant difference in the incidence of grade 3 or higher adverse events between any two treatments. Tislelizumab, combined with the standard first-line chemotherapy regimen, appeared to confer the best progression-free survival (SUCRA = 83.16%), overall survival (SUCRA = 83.16%), and objective response rate (SUCRA = 89%).

**Conclusions:**

Systematic reviews and network meta-analyses integrate evidence from multiple studies, which enables clinicians to make more informed treatment decisions based on comprehensive comparative efficacy and safety data. For patients with RM-NPC, the combination of tislelizumab and chemotherapy is the optimal first-line treatment.

**Systematic review registration:**

https://www.crd.york.ac.uk/PROSPERO/view/CRD42023491570, identifier CRD42023491570.

## Introduction

Nasopharyngeal carcinoma (NPC) is a malignant tumor of the mucous epithelium of the nasopharynx, which often occurs on the top and side walls of the nasopharynx, especially in the pharyngeal recess. NPC is highly invasive, metastatic, and widely prevalent in South China, Southeast Asia, and North Africa. It reveals population susceptibility with notable regional clustering, racial susceptibility, high familial tendency, and a relatively stable incidence rate. Advancements in medical imaging and radiotherapy have increased the local control rate of NPC to 90%, and the 5-year survival rate for newly treated patients exceeds 80%. However, 7.4% of the patients still face local recurrence, and 17.4% suffer from distant metastasis (1–3). Before 2016, fluorouracil and cisplatin were widely used as a first-line treatment for patients with recurrent or metastatic nasopharyngeal carcinoma (RM-NPC) (4). With continued exploration in clinical trials, the current standard first-line treatment regimen for patients with RM-NPC has now become gemcitabine plus cisplatin regimen (5), which has continued to substantially improve patient survival in recent decades. Incorporating a programmed cell death protein-1 (PD-1) inhibitor into the treatment regimen has better therapeutic outcomes, extending the median progression-free survival (PFS) by 9.6 to 11.7 months (6–8). This has become a priority in consideration of new first-line treatment regimens. In addition, studies evaluating PD-1 inhibitors have also demonstrated some salvage therapeutic value in second-line or multi-line therapy, with a single-drug efficacy rate of 20%-30% (9, 10). However, when it comes to second-line treatment, although PD-1 monotherapy has a better safety profile, two RCTs (11, 12) showed that PD-1 monotherapy failed to significantly prolong patients’ survival time compared with chemotherapy. For patients who cannot tolerate or refuse chemotherapy, PD-1 monotherapy can still be considered a viable treatment option. Based on the results of three clinical studies (13–15), PD-1 monotherapy is the preferred treatment option for RM-NPC in third-line and later-line treatments.

For NPC with an initial diagnosis of distant metastasis, traditional views hold that radiotherapy is only used for local symptom control, and there is insufficient evidence that radical radiotherapy for the primary tumor and regional lymph nodes can improve overall survival (OS). However, recent studies have shown that, in patients with low metastatic burden who are sensitive to chemotherapy, the combination of radiotherapy and chemotherapy can significantly improve OS, with some cases even achieving curative outcomes (16, 17). Therefore, it is recommended to determine whether to administer local radiotherapy based on the tumor’s response to chemotherapy, which helps identify patients most likely to benefit while avoiding ineffective treatment for others.

In current clinical practice, first-line treatment options for RM-NPC are trending toward diversification. Moving beyond traditional single-agent chemotherapy, innovative strategies that combine chemotherapy with PD-1 inhibitors have gradually been introduced, and more recently, comprehensive treatment plans incorporating both chemotherapy and local radiotherapy have emerged. Regarding immunotherapy, immune checkpoint inhibitors (ICIs) such as camrelizumab, toripalimab, and tislelizumab have been widely adopted. However, despite the array of available treatment options, the clinical decision-making process is hampered by the lack of direct head-to-head comparative studies. Consequently, physicians face challenges in selecting the optimal treatment based on existing evidence, which not only impacts treatment efficiency but may also adversely affect patient prognosis. In this context, this study employs a Bayesian network meta-analysis to systematically compare and evaluate various treatment strategies for RM-NPC, thereby providing high-quality evidence to support clinical practice and optimize decision-making pathways.

## Methods

We conducted this meta-analysis in accordance with the Preferred Reporting Items for Systematic Reviews and Meta-Analyses (PRISMA). The Bayesian approach facilitates the indirect evaluation of treatments that have not been tested in parallel, offering a simplified method for probabilistic assertions and predicting the efficacy and safety of treatment regimens (18). The protocol was registered in the Prospective Register of Systematic Reviews (PROSPERO) (CRD42023491570).

### Data sources and searches

We conducted a systematic search of RCTs in PubMed, Embase, Web of Science, Cochrane Library, and ClinicalTrials.gov from inception until March 1, 2024. The principal search keywords were “first-line” and “nasopharyngeal carcinoma (NPC),” with the inclusion criteria limited to randomized controlled trials. Conference abstracts released as of March 1, 2024, were also sourced from renowned scientific organizations, including the American Society of Clinical Oncology (ASCO), the European Society of Medical Oncology (ESMO), and the Chinese Society of Clinical Oncology (CSCO), to enrich the research compilation. The comprehensive search methodology is described in **Supplementary Table 1**, providing a structured overview of the investigative parameters.

### Inclusion criteria

The criteria encompass published phase 2/3 randomized clinical trials, ensuring a comprehensive inclusion of relevant studies. Inclusion criteria are:

1. Pathologically confirmed NPC with primary metastatic disease (according to American Joint Committee on Cancer Staging System for NPC, 8th edition) or local recurrence after curative radiotherapy.
2. Comparison of any two or more arms of first-line treatments for patients with RM-NPC.
3. Trials that presented outcomes based on at least one of the following clinical measurements:

PFS refers to the period between the start of treatment and the observation of disease progression or death due to any reason. OS refers to the time from randomization to death, regardless of the cause of death. Objective response rate (ORR) is defined as the proportion of patients achieving objective response. Adverse events (AEs) of grade 3 or higher were defined and graded according to the National Cancer Institute Common Terminology Criteria for AEs.

### Exclusion criteria

Clinical studies mainly focused on patients with locally advanced NPC and analyzed RM-NPC as a subgroup.

The aim of the study was to compare maintenance therapy with optimal life support therapy.

Initially, we screened titles and abstracts of all retrieved articles, then conducted a thorough review and assessment of the full text of potentially eligible articles based on predefined inclusion and exclusion criteria. The selected articles should include data from various study periods and follow-up durations, including the latest information derived from mature or long-term follow-ups of the original research.

### Data extraction and risk-of-bias assessment

Two authors (Cai and Lin) independently reviewed all eligible studies and extracted information into a spreadsheet, including study name, study phase, year of publication, first author, number of patients included, patient characteristics (e.g., number of female and male patients, EBV DNA level, performance status), treatment regimens, and clinical outcomes. We prioritized survival data assessed by independent committees over data assessed by researchers.

We evaluated the risk of bias in individual studies using the Cochrane Risk of Bias Tools (RoB-2), which is based on the following domains: risk of bias from the randomization process, risk of bias due to deviations from the intended interventions, risk of bias from missing outcome data, risk of bias from measurement of the outcome, and risk of bias from selection of the reported result (19). If there are any objections, the research team will jointly review to reach a consensus.

### Data synthesis statistical analysis

In our study, we integrated direct and indirect evidence to evaluate the effectiveness and safety profiles of various treatments. We presented the hazard ratios (HR) for survival indicators, including PFS and OS, alongside the odds ratios (OR) for binary outcomes such as the ORR and incidence of grade 3 or higher AEs. Each indicator was accompanied by a 95% confidence interval (CI). For network meta-analysis, we used Stata (version 18.0) to generate a network graph illustrating the direct and indirect comparison relationships between treatment regimens with different outcomes for different patient populations in the trial (20).

Statistical analyses were conducted using the R software (version 4.3.1) with the ‘gemtc’ package. We performed pairwise meta-analyses of trials with the same treatment modalities and employed a Bayesian fixed-effect consistency model to compare different approaches. Markov Chain Monte Carlo (MCMC) sampling was performed using JAGS software (version 4.3.1) with 50,000 simulations. The first 20,000 simulations were treated as the burn-in period, and a thinning interval of 1 was applied. By analyzing the trace plots, we can determine whether each MCMC sample has reached stability and achieved sufficient overlap during the computational process, thereby assessing the degree of model convergence (21). The functions of the density plots and the Brooks-Gelman-Rubin diagnostic plots were consistent with those of the trace plots, both of which were used to diagnose the degree of model fit but in different ways. Once convergence was established, the posterior analysis of the model parameters was presented as the output of the network meta-analysis.

Network meta-analysis estimates the overall ranking of each process by calculating the surface under the cumulative ranking curve (SUCRA) of each treatment regimen. The value of SUCRA ranges from 0 and 1. A SUCRA value of 1 indicates that the intervention is effective, whereas 0 indicates that the intervention is ineffective (22). The quality of interventions can be ranked based on the SUCRA value (23). We used the I^2^ statistic to assess heterogeneity between studies, where heterogeneity was considered low, moderate, or high if I^2^ values were less than 25%, between 25% and 50%, and greater than 50%, respectively (24).

## Results

### Systematic review and characteristics

**Figure 1:**
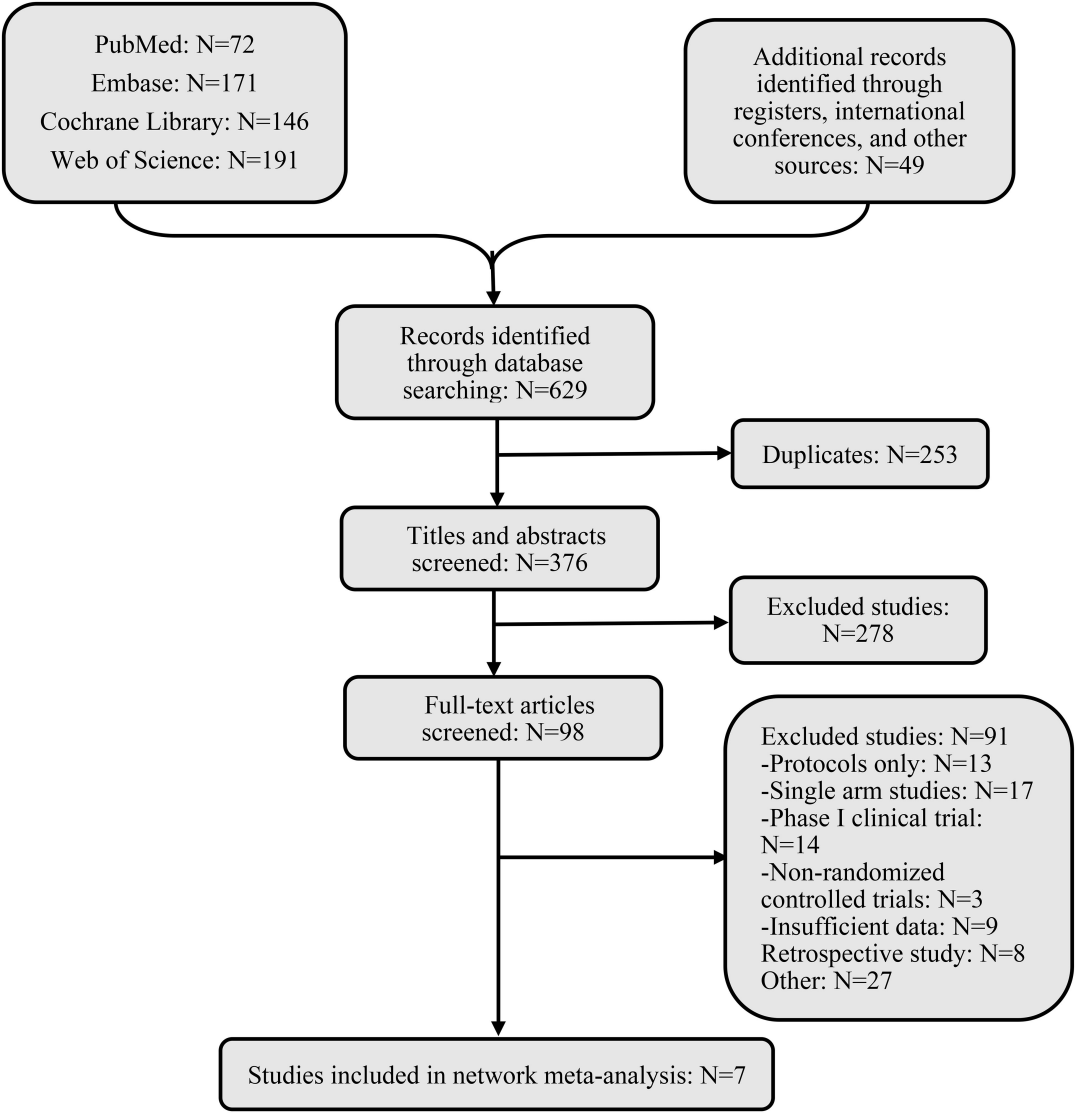
Flow chart of literature screening.

**Figure 2:**
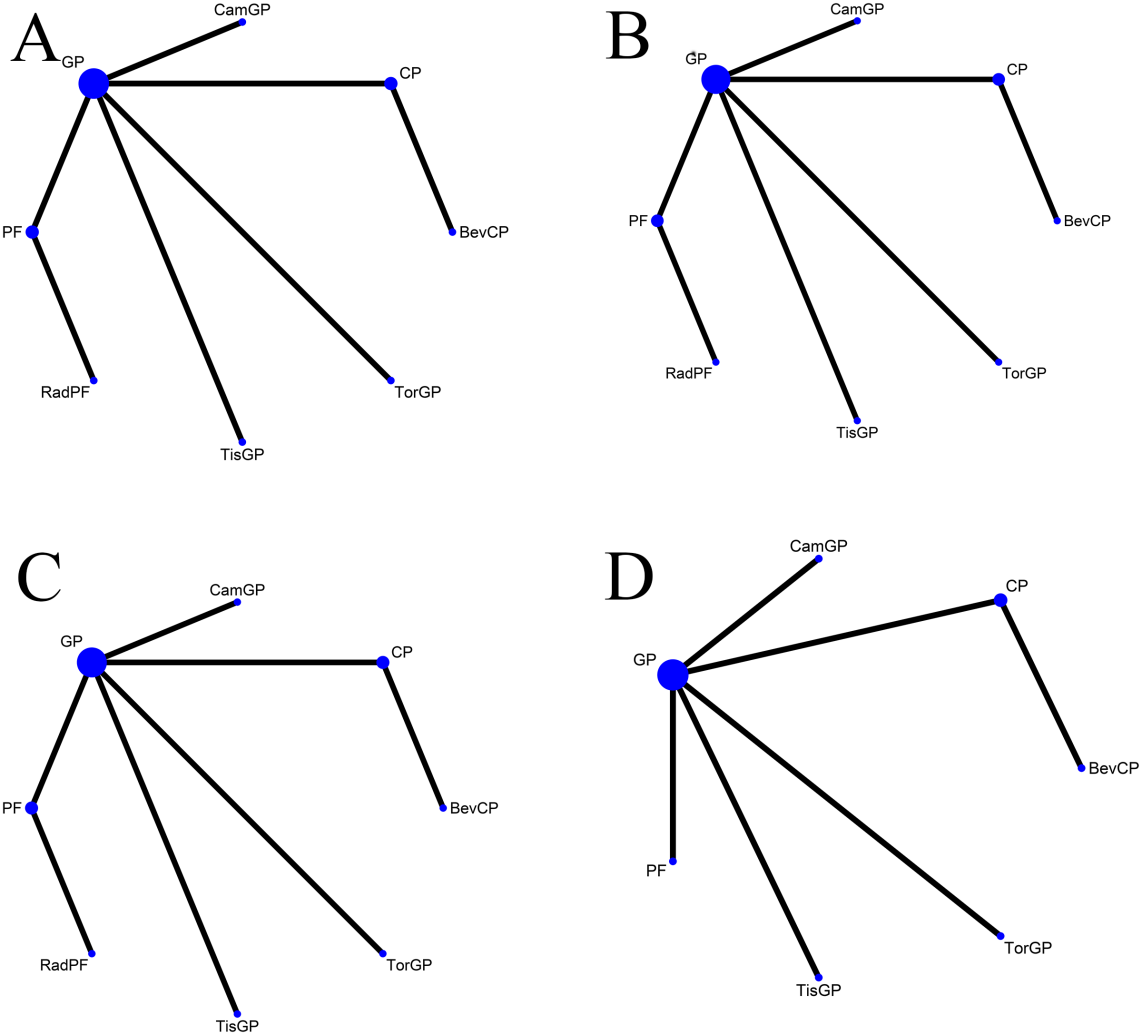
Network diagram Network meta-analysis of comparisons across different outcomes of first-line treatments in patients with RM-NPC. **(A)** Comparison of overall survival. **(B)** Comparison of progression-free survival. **(C)** Comparison of objective response rate. **(D)** Comparison of grade 3 or more adverse events. GP, gemcitabine plus cisplatin; PF, fluorouracil plus cisplatin; CP, platinum plus taxanes; TisGP, tislelizumab plus GP; TorGP, toripalimab plus GP; CamGP, camrelizumab plus GP; RadPF, radiotherapy plus PF; BevCP, bevacizumab plus CP.

**Figure 3:**
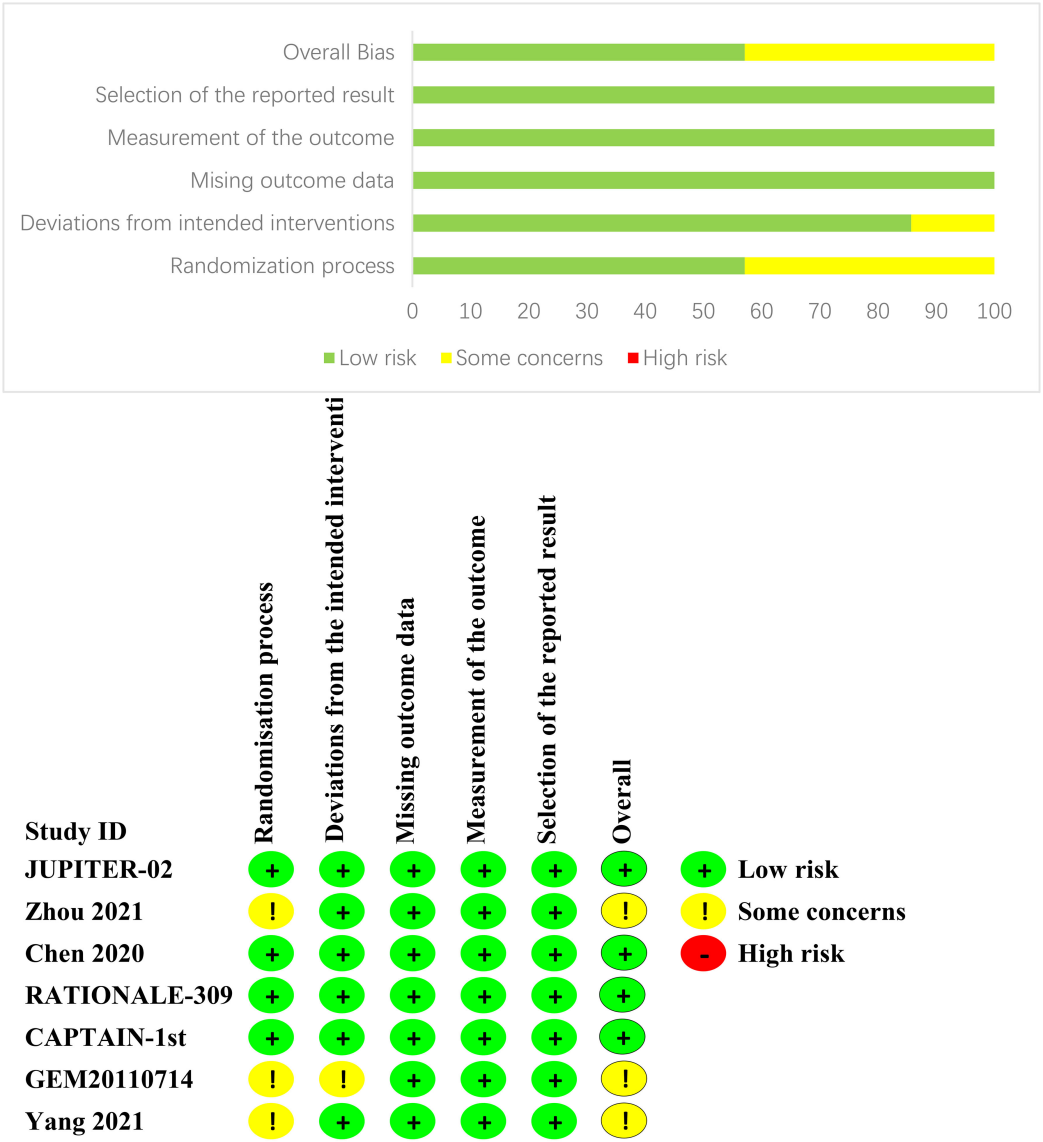
Risk of bias assessment.

After an exhaustive database search, we initially screened 629 articles. Subsequently, 98 articles were considered suitable for in-depth evaluation after reviewing their titles and abstracts. The meta-analysis was conducted using seven pertinent studies (**Figure 1**), which included 1495 patients who underwent eight different treatment regimens. These included platinum-based chemotherapy regimens such as fluorouracil with cisplatin (PF), gemcitabine with cisplatin (GP), and innovative treatments combining platinum drugs with taxanes (CP). Additionally, the analysis considered PD-1 inhibitors in combination with chemotherapy, featuring ICIs such as toripalimab (TorGP), tislelizumab (TisGP), and camrelizumab (CamGP). Other treatment regimens evaluated were chemotherapy paired with bevacizumab (BevCP) and the synergistic use of chemotherapy and radiotherapy (RadPF). The network diagrams are presented in **Figure 2**. The main characteristics of all studies are reported in **Table 1**. **Figure 3** summarises the detailed risk of bias assessments across all included studies.

**Table 1:** Literature search strategy.

Search Strategy in PubMed
((((((((first-line[Title/Abstract]) OR (untreated[Title/Abstract])) OR (treatment naive[Title/Abstract])) OR (chemo naive[Title/Abstract])) OR (front line[Title/Abstract])) OR (first line[Title/Abstract])) OR (1st line[Title/Abstract])) OR (1st-line[Title/Abstract])) AND ((((((((randomized controlled trial[Title/Abstract]) OR (RCT[Title/Abstract])) OR (controlled clinical trial[Title/Abstract])) OR (randomized[Title/Abstract])) OR (randomly[Title/Abstract])) OR (trial[Title/Abstract])) OR (placebo[Title/Abstract])) AND ((((metastatic[Title/Abstract]) OR (advanced[Title/Abstract])) OR (recurrent[Title/Abstract])) AND (((((Carcinoma, Nasopharyngeal[Title/Abstract]) OR (Carcinomas, Nasopharyngeal[Title/Abstract])) OR (Nasopharyngeal Carcinomas[Title/Abstract])) OR (NPC[Title/Abstract])) OR ("Nasopharyngeal Carcinoma"[Mesh]))))

### Network meta-analysis in RM-NPC

The network meta-analysis included PFS, OS, and ORR for all treatment regimens, as well as grade 3 or higher AEs for 7 treatments.

**Figure 4:**
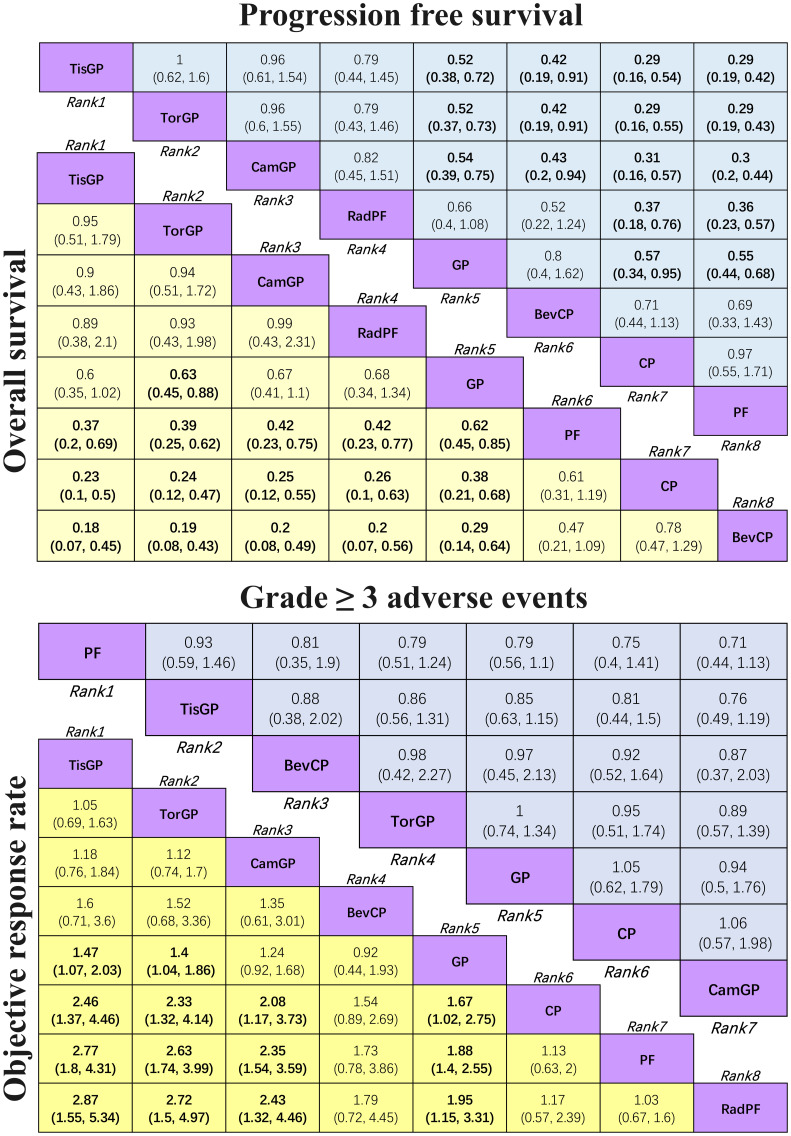
League table for network meta-analysis Efficacy and safety profiles of the network meta-analysis in patients with RM-NPC. In the case of HR and 95% CI for overall survival and progression-free survival, an HR of less than 1.00 indicates better survival benefits. In the case of OR and 95% CI for objective response rate, an OR > 1.00 indicates a better efficacy. In the case of OR and 95% CI for grade ≥ 3 adverse events, an OR < 1.00 indicates better safety.

In terms of PFS (**Figure 4**), patients who received PD-1 inhibitor combinations were more likely to obtain greater PFS benefits than those who received chemotherapy alone. Specifically, TisGP (HR = 0.52, 95% CI: 0.38–0.72), TorGP (HR = 0.52, 95% CI: 0.37–0.73), and CamGP (HR = 0.54, 95% CI: 0.39–0.75) all showed significantly lower HR compared with GP, indicating that the combination of PD-1 inhibitors with chemotherapy provides a substantial advantage in extending PFS. RadPF demonstrated a significant improvement in PFS compared with PF (HR = 0.36, 95% CI: 0.23–0.57). However, when compared with GP, RadPF did not yield a statistically significant difference in PFS (HR = 0.66, 95% CI: 0.40–1.08). Furthermore, GP demonstrated superior PFS compared to both CP (HR = 0.57, 95% CI: 0.34–0.95) and PF (HR = 0.55, 95% CI: 0.44–0.68).

In terms of OS (**Figure 4**), TorGP was the only regimen associated with a significant improvement in OS compared to standard chemotherapy (HR = 0.63, 95% CI: 0.45–0.88). While TisGP (HR = 0.60, 95% CI: 0.35–1.02) and CamGP (HR = 0.67, 95% CI: 0.41–1.10) showed trends toward improved OS, neither reached statistical significance. In addition, when compared to PF (HR = 0.62, 95% CI: 0.45–0.85) and CP (HR = 0.38, 95% CI: 0.21–0.68), the GP regimen demonstrated a significant improvement in OS in patients with RM-NPC.

Regarding ORR (**Figure 4**), both TisGP (OR = 1.47, 95% CI: 1.07–2.03) and TorGP (OR = 1.4, 95% CI: 1.04–1.86) were more likely to achieve a higher response rate compared to standard chemotherapy, whereas CamGP (OR = 1.24, 95% CI: 0.92–1.68) did not reach statistical significance. Similarly, when compared to CP (OR = 1.67, 95% CI: 1.02–2.75) and PF (OR = 1.88, 95% CI: 1.40–2.55), GP significantly improved ORR, which is consistent with the observed benefits in PFS and OS. Furthermore, GP showed a notable improvement in ORR compared to RadPF (OR = 1.95, 95% CI: 1.15–3.31). BevCP ranked second only to PD-1 inhibitor combinations in terms of ORR, although it did not differ significantly from the two-drug chemotherapy regimen.

The safety profile assessment (**Figure 4**) revealed that there were no significant differences in the incidence of grade ≥ 3 AEs among the treatment regimens evaluated: PF, GP, CP, TisGP, TorGP, CamGP, and BevCP. Notably, combinations involving PD-1 inhibitors were not associated with significant increase in grade ≥3 treatment-related AEs. However, immune-related adverse events (irAEs), which are not typically observed in chemotherapy alone, did occur in patients receiving these immunotherapeutic regimens, namely PD-1 inhibitors. These findings suggest that the addition of PD-1 inhibitors to standard chemotherapy maintains an acceptable safety profile while introducing distinct adverse event patterns characteristic of immune checkpoint inhibition.

### Rank probabilities

**Figure 5:**
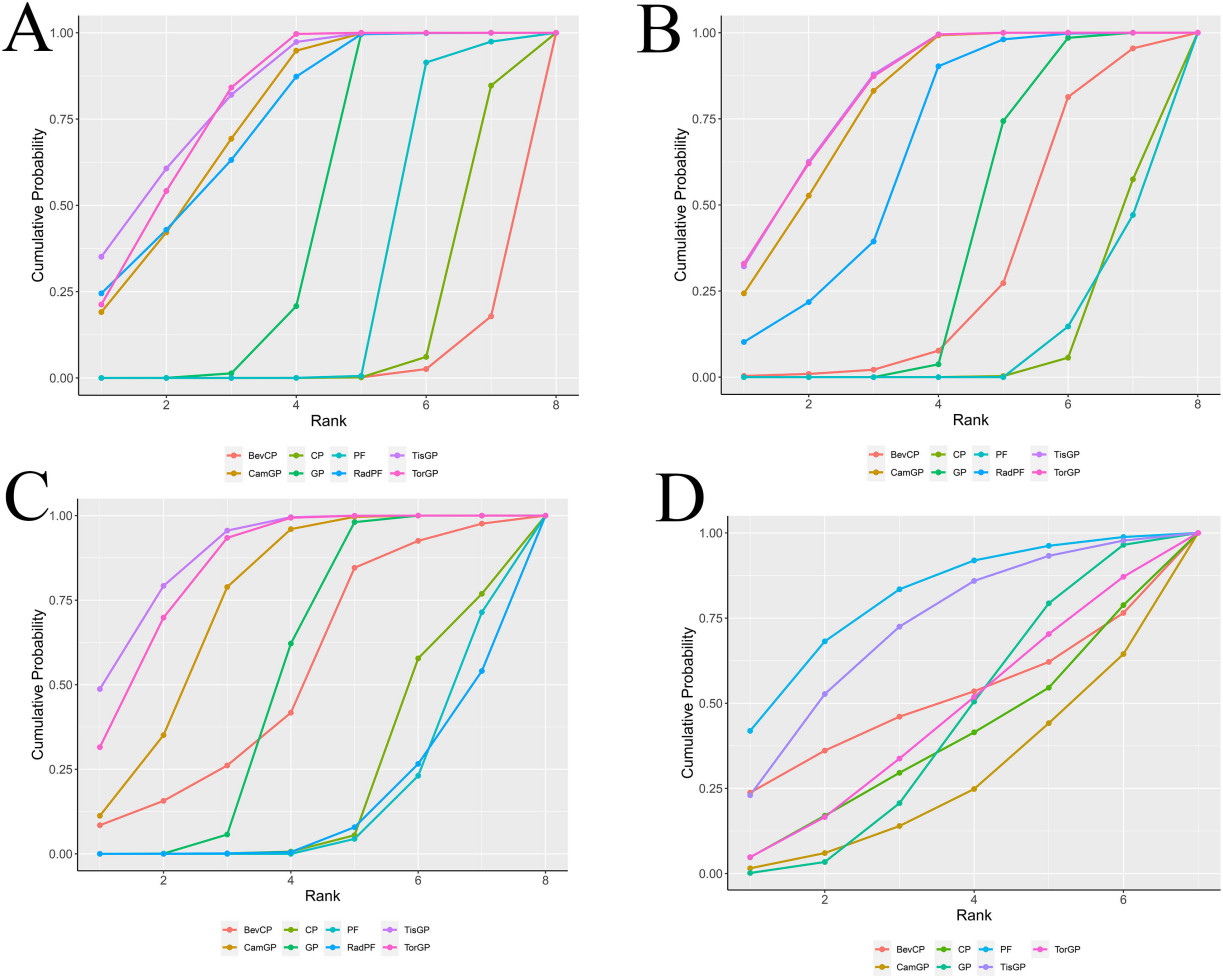
Surface under the cumulative ranking curve plot cumulative ranking probabilities of treatment. The surface under the cumulative ranking curve (SUCRA) value represents the probability that each treatment has of being among the best in the network, with larger values indicating higher ranking probabilities. The profiles indicate the probability of each first-line treatment being ranked from first to last on overall survival **(A)**, progression-free survival **(B)**, objective response rate **(C)**, and grade ≥ 3 adverse events **(D)**.

For patients with RM-NPC (**Figure 5**), TisGP demonstrated the highest likelihood of ranking first in PFS (SUCRA = 83.16%), OS (SUCRA = 82.15%), and ORR (SUCRA = 89%). TorGP ranked second in efficacy across all three endpoints, with SUCRA values of 83.12% for PFS, 79.89% for OS, and 84.87% for ORR. Similarly, CamGP achieved good therapeutic outcomes, ranking third in PFS (SUCRA = 79.91%), OS (SUCRA = 75.03%), and ORR (SUCRA = 74.41%). PF exhibited the lowest toxicity profile, ranking first in terms of grade ≥ 3 adverse events (SUCRA = 80.08%).

### Subgroup analysis

**Figure 6:**
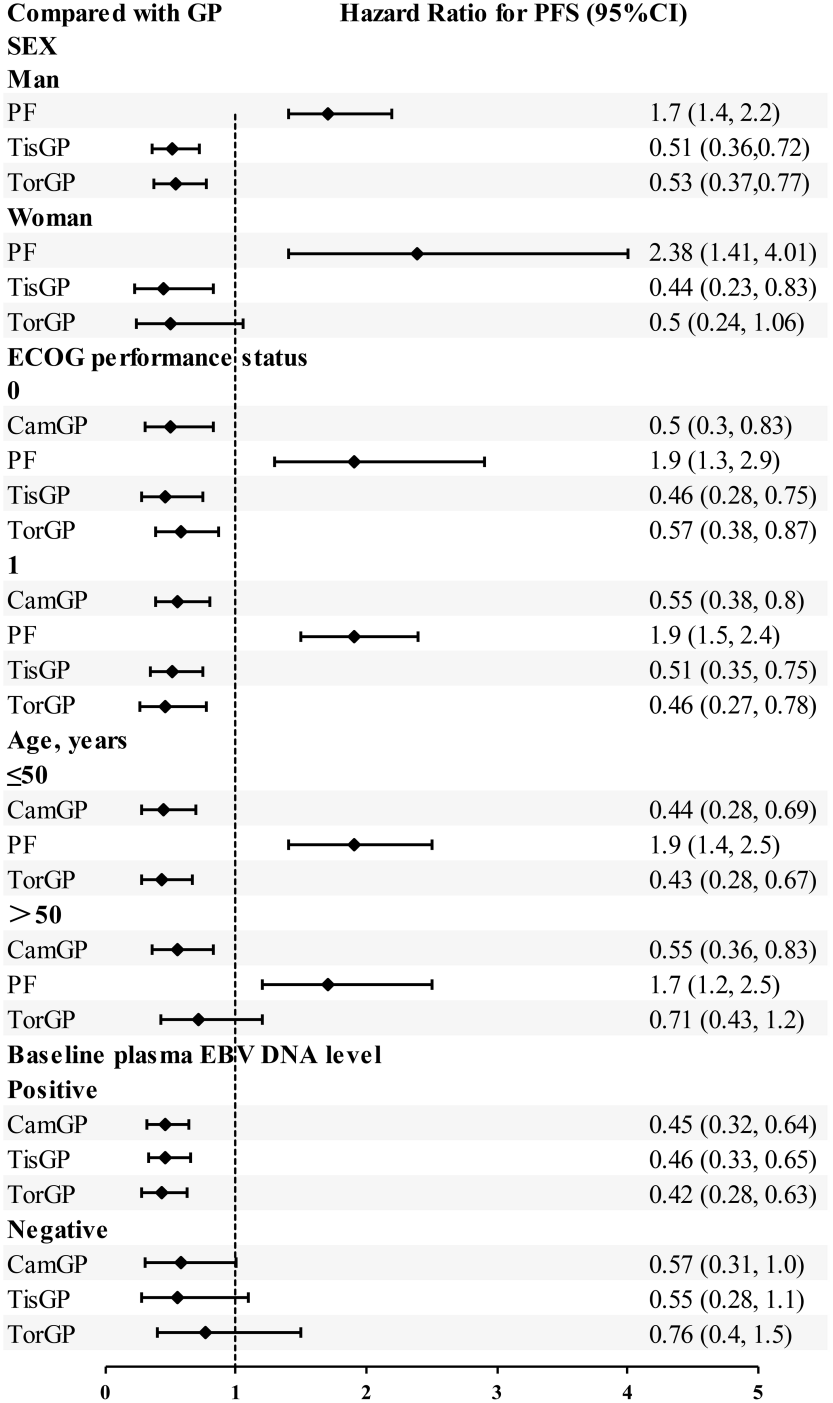
Subgroup analysis of progression-free survival.

**Figure 7:**
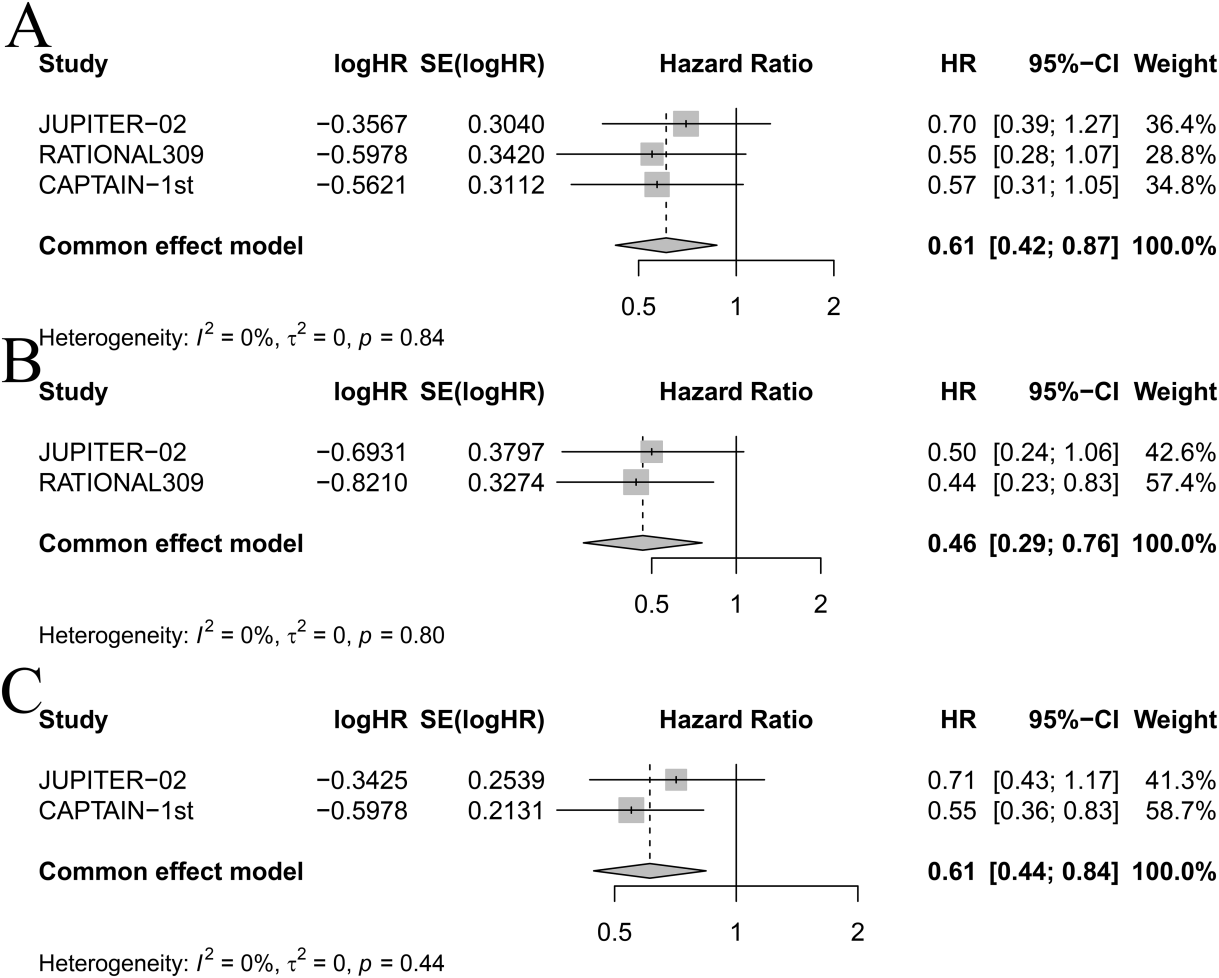
Forest plot for **(A)** progression-free survival in patients with negative EBV DNA expression, **(B)** progression-free survival in females, and **(C)** progression-free survival in patients over 50 years old.

Based on EBV DNA levels (**Figure 6**), considering the clinical outcomes of RM-NPC patients with varying levels of EBV DNA expression, only PFS could be estimated in the subgroup analysis. Patients were categorized into two subgroups based on their EBV DNA status: negative group and positive group. In patients with positive EBV DNA expression, PD-1 inhibitor combinations showed superior outcomes compared to GP. Specifically, the HR values for CamGP, TisGP, and TorGP were 0.45 (95% CI: 0.32–0.64), 0.46 (95% CI: 0.33–0.65), and 0.42 (95% CI: 0.28–0.63), respectively. In contrast, in patients with negative EBV DNA expression, PD-1 inhibitor combinations showed a beneficial trend compared to chemotherapy alone, though the difference did not reach statistical significance. However, when the effect sizes from the three studies were pooled (**Figure 7A**), the combined analysis achieved statistical significance, with a pooled HR of 0.61 (95% CI: 0.42–0.87), and no heterogeneity was observed (I² = 0%).

A subgroup analysis based on performance status (PS) scores (**Figure 6**) was conducted among patients with PS scores of 0–1 across five treatment groups: CamGP, TisGP, TorGP, GP, and PF. In patients with a PS of 0, the PD−1 inhibitor combinations demonstrated superior efficacy relative to GP. Specifically, TisGP achieved a hazard ratio (HR) of 0.46 (95% CI: 0.28–0.75), CamGP an HR of 0.50 (95% CI: 0.30–0.83), and TorGP an HR of 0.57 (95% CI: 0.38–0.87). Similarly, among patients with a PS of 1, PD−1 inhibitor combinations outperformed GP, with TorGP registering an HR of 0.46 (95% CI: 0.27–0.78), TisGP an HR of 0.51 (95% CI: 0.35–0.75), and CamGP an HR of 0.55 (95% CI: 0.38–0.80). Overall, the subgroup analysis clearly indicates that, for patients with PS scores of 0 and 1, PD−1 inhibitor combinations offered superior efficacy compared to GP.

Among female patients (**Figure 6**), compared with GP, TisGP (HR = 0.44, 95% CI: 0.23–0.83) provided a significantly better PFS benefit, whereas TorGP did not show significantly improved efficacy compared to GP. After combining the effect sizes of the two studies (**Figure 7B**), the pooled results were statistically significant (pooled HR: 0.46, 95% CI: 0.29–0.76, I² = 0%). In male patients, both TisGP (HR = 0.51, 95% CI: 0.36–0.72) and TorGP (HR = 0.53, 95% CI: 0.37–0.77) appeared to offer superior efficacy compared to GP. Overall, the gender subgroup analysis indicated that, relative to GP, TisGP provided better PFS benefits in both female and male patients, while the advantage of TorGP was observed only in male patients.

In the subgroup analysis of patients aged ≤ 50 years (**Figure 6**), both TorGP (HR = 0.43, 95% CI: 0.28–0.67) and CamGP (HR = 0.44, 95% CI: 0.28–0.69) demonstrated significant improvements in PFS compared to the GP regimen. For patients over 50 years old, CamGP (HR = 0.55, 95% CI: 0.36–0.83) was associated with improved PFS, while TorGP showed a trend toward benefit (HR = 0.71, 95% CI: 0.43–1.20) that did not reach statistical significance. After pooling the effect estimates from the two studies (**Figure 7C**), the results achieved statistical significance (pooled HR: 0.61, 95% CI: 0.44–0.84, I² = 0%). These findings indicate that patients with RM-NPC derive PFS benefits from the combination of PD-1 inhibitors and chemotherapy, regardless of their age.

## Discussion

### Principal findings

In this systematic review and network meta-analysis, we comprehensively evaluated the comparative efficacy and safety of various first-line treatment options, including all available PD-1 inhibitors and their combination strategies, for patients with RM-NPC. The results suggest that:

1. Combination of chemotherapy and PD-1 inhibitors has demonstrated significant improvements in OS, PFS, and ORR compared to chemotherapy alone, chemotherapy combined with anti-angiogenic therapy, or chemotherapy combined with radiotherapy.
2. TisGP presented the best PFS, OS, and ORR for patients with RM-NPC.
3. In patients with RM-NPC, RadPF demonstrated a trend toward improvement in PFS and OS compared to GP, although it did not reach statistical significance.
4. Adding PD-1 inhibitors or antiangiogenic drugs to standard chemotherapy did not significantly increase toxicity.

Due to the unique immune substrates of NPC, including abundant lymphocyte infiltration, high expression of PD-1, and the presence of multiple immune targets (CD40, CD70, CD80, and CD86), the inclusion of ICIs, for example PD-1 inhibitors in the treatment of NPC has a strong biological basis (25). NPC is primarily associated with EBV infection, which provides additional immune susceptibility factors through the expression of EBV antigen and CD4+/CD8+ cell target proteins. Accumulating evidence highlights the importance of plasma EBV DNA in diagnosing patients with NPC. Baseline and longitudinal evaluations of plasma EBV DNA during induction chemotherapy have consistently demonstrated a robust prognostic value for locally advanced disease. In the POLARIS-02 study using toripalimab (13), a rapid decrease in plasma EBV DNA copy number was associated with a positive prognosis in patients with metastatic NPC. From the results of our data analysis, it is evident that regardless of the baseline copy number of EBV DNA, PD-1 inhibitors combined with chemotherapy demonstrate better efficacy than chemotherapy alone. However, the benefits were more pronounced when the baseline copy number of EBV DNA was higher. Although EBV-induced LMP1 and the IFN-γ pathway jointly regulate PD-L1 expression in NPC cells, with PD-L1 expression being higher in EBV-positive NPC cells than in EBV-negative NPC cells (26), no correlation was found between PD-L1 expression levels and plasma EBV DNA copy numbers (27). Therefore, further research is needed to explore the underlying mechanisms through which RM-NPC patients with high baseline EBV copies may benefit from ICIs.

Anti-angiogenic drugs can act on the tumor microenvironment, causing the degradation of existing tumor blood vessels and inhibiting the formation of new blood vessels within the tumor. Bevacizumab is a recombinant humanized monoclonal antibody that targets VEGF, playing a crucial role in the treatment of various malignant tumors and expanding into other fields. While the combined use of bevacizumab with chemotherapy did not improve OS or PFS in patients with RM-NPC, it demonstrated a notable improvement in ORR, second only to the combination of PD-1 inhibitors and chemotherapy. Bevacizumab also exhibited good safety and tolerability. Given its favorable toxicity profile and tumor reduction rate, bevacizumab combined with chemotherapy can be considered as an option for NPC patients with heavy tumor burden or pursuing short-term efficacy, such as in neoadjuvant or concurrent chemotherapy settings (28). Although previous studies and theoretical considerations have suggested that combining bevacizumab with chemotherapy might have certain therapeutic advantages, the results section of this study did not directly confirm this. However, the evaluation of bevacizumab in combination with paclitaxel introduces potential confounding factors, as the observed outcomes may be attributed to either agent or their synergistic effect. Additionally, comparisons with non-standard chemotherapy regimens introduce further uncertainty that may result from differences in patient selection criteria or treatment protocols. Future studies should consider isolating the effects of bevacizumab through RCTs with standardized regimens and clear patient stratification to minimize such biases.

For newly diagnosed advanced NPC with metastasis, local radiotherapy has the significance of prolonging survival. However, in the era of standard treatment with GP, the importance of local radiotherapy still needs further research. According to the results of this study, it is evident that RadPF exhibited a beneficial trend in OS and PFS compared to GP, with a more favorable efficacy ranking. The combination of radiotherapy with GP or radiotherapy combined with ICIs based on standard chemotherapy is a promising direction for future clinical research exploration. Chemotherapy combined with PD-1 inhibitors and radiotherapy has been explored in patients with locally advanced NPC. Studies have found that the addition of sintilimab to radiotherapy and chemotherapy can improve event-free survival (EFS) despite having high but controllable adverse events (29).

Based on our findings in the network meta-analysis, the three first-line chemo-immunotherapies demonstrated notable improvements in PFS, OS, and ORR compared to GP. Among the three chemotherapy plus PD−1 inhibitor combination groups, TisGP showed the most favorable trends in PFS, OS, and ORR benefits; however, no significant statistical differences were observed, which should be interpreted with caution. Additionally, TorGP was the only PD-1 inhibitor proven to improve OS compared to standard chemotherapy, with a statistically significant difference. All subgroup analyses yielded consistent conclusions, indicating that the entire population of patients with NPC has the potential to benefit from PD-1 inhibitors combined with chemotherapy. When comparing the treatment efficacy of the three PD-1 inhibitors, the following points should be noted. First, there are significant differences in disease characteristics and baseline conditions among the patient populations enrolled in different studies, which directly affect the comparability of efficacy evaluations. For example, in the CAPTAIN-1ST study, the enrolled patients had poorer baseline characteristics—65% had a PS score of 1, and all patients had distant metastases, with 65% of subjects being recurrent with distant metastasis; patients with only locoregional recurrence were not included. In the JUPITER-02 study, 60% of the enrolled patients had recurrent NPC, while 40% were patients with *de novo* distant metastasis. In contrast, in the RATIONALE-309 study, nearly 96.2% of the subjects were initially diagnosed with metastatic disease, with only 3.8% being recurrent patients. These differences in patient populations may limit the comparability of the efficacy evaluation results. Second, the PS score is an important indicator for assessing a patient’s physical condition, and differences in the distribution of PS scores across studies may significantly affect the overall efficacy evaluation outcomes. Finally, the PD-L1 expression level is a crucial factor influencing efficacy. Previous studies, such as CAPTAIN and POLARIS-02, have shown that patients with high PD-L1 expression in NPC tend to have better treatment outcomes with PD-1 inhibitors compared to those with low expression. For instance, in the RATIONALE-309 study, the proportion of subjects with PD-L1 ≥10% was relatively high, which may lead to better outcomes in the efficacy evaluations. Therefore, when comparing these three studies, it is essential to fully consider the aforementioned factors to ensure the accuracy and comparability of the efficacy assessments.

### Implication

By integrating evidence from RCTs, this review provides a reference source for clinicians to evaluate the advantages and disadvantages of practical choices among various promising options. Combining PD-1 inhibitors with standard chemotherapy can significantly improve the efficacy of RM-NPC without increasing the incidence of new AEs, making it a new standard treatment regimen. Compared with standard chemotherapy, RadPF appeared to offer benefits in terms of OS and PFS. Based on the above research findings, future trial designs should prioritize investigating the efficacy and safety of GP combined with radiotherapy, as well as chemo-immunotherapy combined with radiotherapy. Furthermore, additional head-to-head clinical trials should be conducted to compare the clinical benefits and safety of combination therapy with these PD-1 inhibitors.

### Limitations

First, given the limited number of studies that meet our inclusion criteria, including those with small sample sizes, this may increase the risk of bias, affect statistical power, neglect heterogeneity, and potentially lead to an overinterpretation of the analysis outcomes.

Second, the baseline characteristics of patients are not consistent. For example, in the study of chemotherapy combined with radiotherapy, all patients included were initially diagnosed with metastatic NPC and did not include recurrent NPC. In addition, the included patients were those who achieved complete or partial remission after three cycles of chemotherapy.

Finally, to facilitate the comparison of the efficacy of multiple treatment options, we assumed that carboplatin and cisplatin are equally effective, and similarly, paclitaxel and docetaxel are assumed to be equally effective. This increases the risk of bias, therefore, the results should be interpreted with caution.

The results of network meta-analysis should be used as one of the references for clinical decision-making rather than the sole basis. In practical applications, it is necessary to combine other types of evidence, such as the results of RCTs, recommendations from clinical practice guidelines, and patient values and preferences, to make comprehensive decisions. With the emergence of new research evidence, the results of network meta-analysis may change. Therefore, when using network meta-analysis, it is essential to stay up-to-date with the latest research progress in the field and verify and update the analysis results in a timely manner to ensure the scientific and timely nature of clinical decision-making.

In conclusion, combining chemotherapy with PD-1 inhibitors proves more effective than other approaches, including dual-drug chemotherapy alone, chemotherapy plus radiotherapy, and chemotherapy combined with targeted therapy. Furthermore, for patients with RM-NPC, the combination of tislelizumab and chemotherapy appears to be the optimal first-line treatment regimen. Systemic therapy combined with local radiotherapy can enhance the prognosis for patients with newly diagnosed metastatic NPC. It is advisable to determine whether local radiotherapy is necessary based on the tumor’s response following chemotherapy.

## Data Availability

The original contributions presented in the study are included in the article/**Supplementary Material**. Further inquiries can be directed to the corresponding authors.
