# Supplementary material for: Efficacy and safety of first-line treatments for recurrent or metastatic nasopharyngeal carcinoma: a systematic review and network meta-analysis
Source: Front Immunol. 2025 Jun 9;16:1485609. doi: 10.3389/fimmu.2025.1485609 (PMC12183282; doi:10.3389/fimmu.2025.1485609)

Table 1. Literature search strategy.

| **Search Strategy in PubMed** |
| --- |
| ((((((((first-line[Title/Abstract]) OR (untreated[Title/Abstract])) OR (treatment naive[Title/Abstract])) OR (chemo naive[Title/Abstract])) OR (front line[Title/Abstract])) OR (first line[Title/Abstract])) OR (1st line[Title/Abstract])) OR (1st-line[Title/Abstract])) AND ((((((((randomized controlled trial[Title/Abstract]) OR (RCT[Title/Abstract])) OR (controlled clinical trial[Title/Abstract])) OR (randomized[Title/Abstract])) OR (randomly[Title/Abstract])) OR (trial[Title/Abstract])) OR (placebo[Title/Abstract])) AND ((((metastatic[Title/Abstract]) OR (advanced[Title/Abstract])) OR (recurrent[Title/Abstract])) AND (((((Carcinoma, Nasopharyngeal[Title/Abstract]) OR (Carcinomas, Nasopharyngeal[Title/Abstract])) OR (Nasopharyngeal Carcinomas[Title/Abstract])) OR (NPC[Title/Abstract])) OR ("Nasopharyngeal Carcinoma"[Mesh])))) |

Figure 1. Convergence of the three Markov Chain Monte Carlo (MCMC) chains established by of the history feature for overall survival (A), progression-free survival (B), objective response rate (C), and grade ≥3 AEs (D).

A History for overall survival


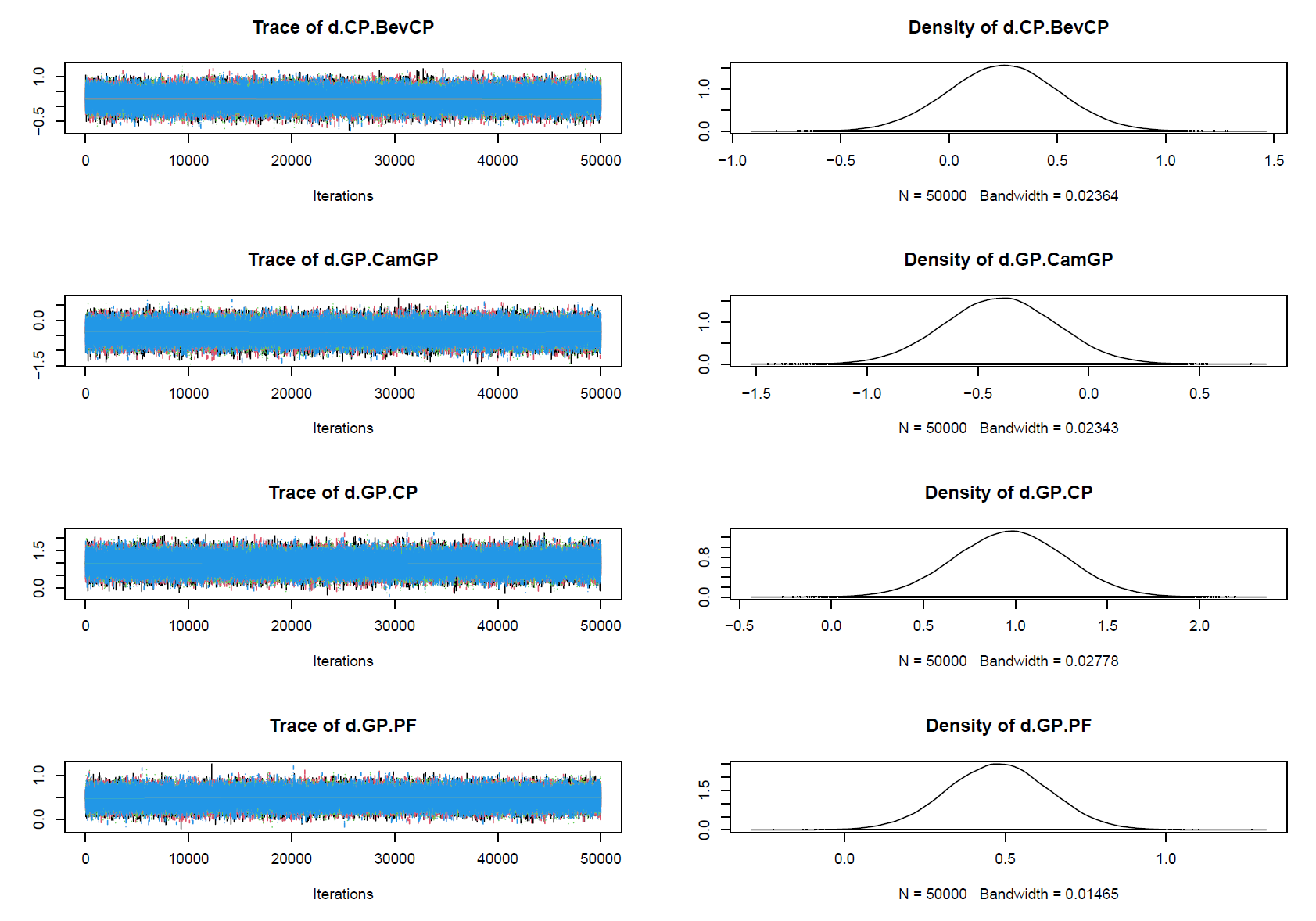


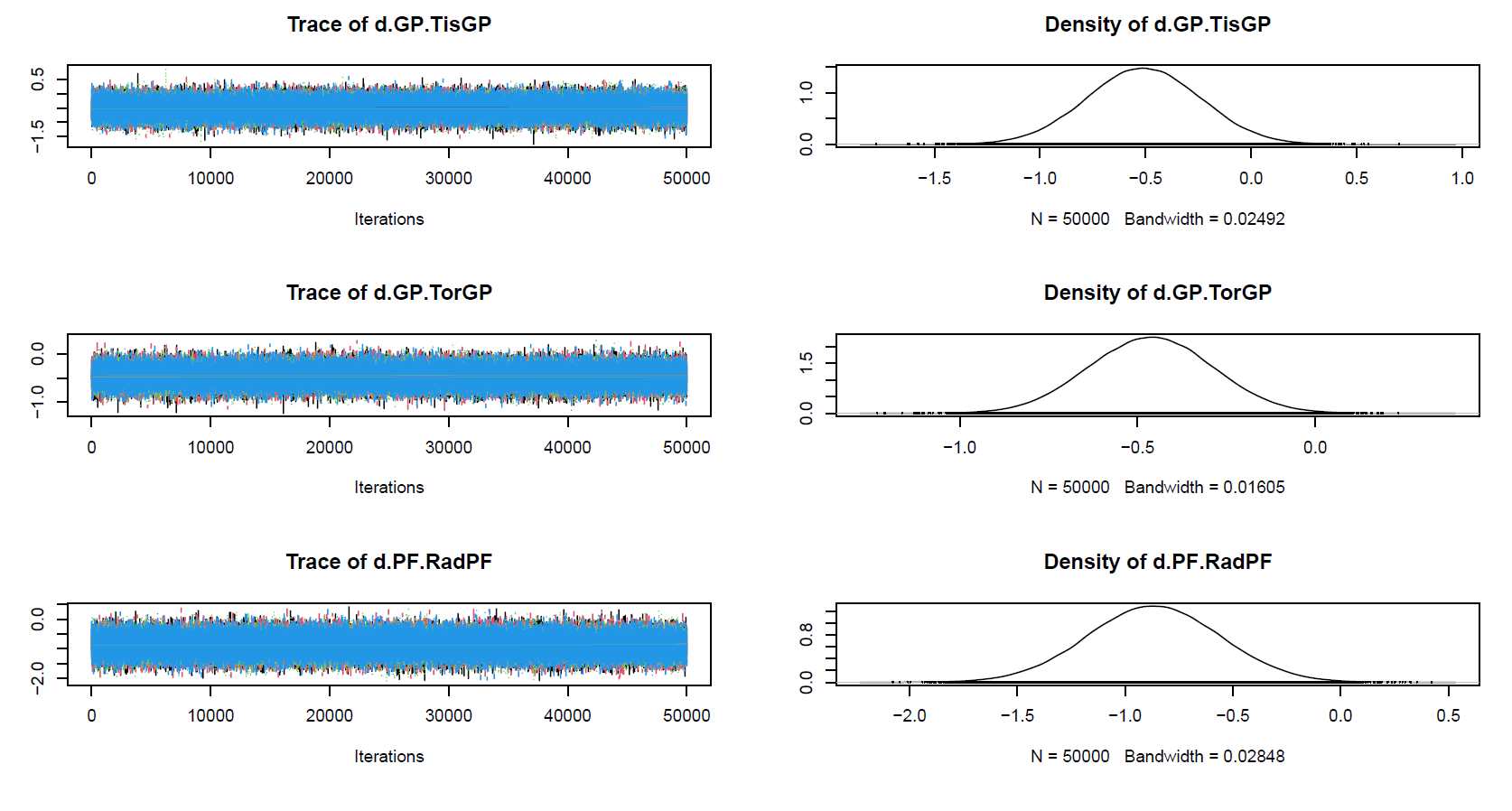


B History for progression-free survival


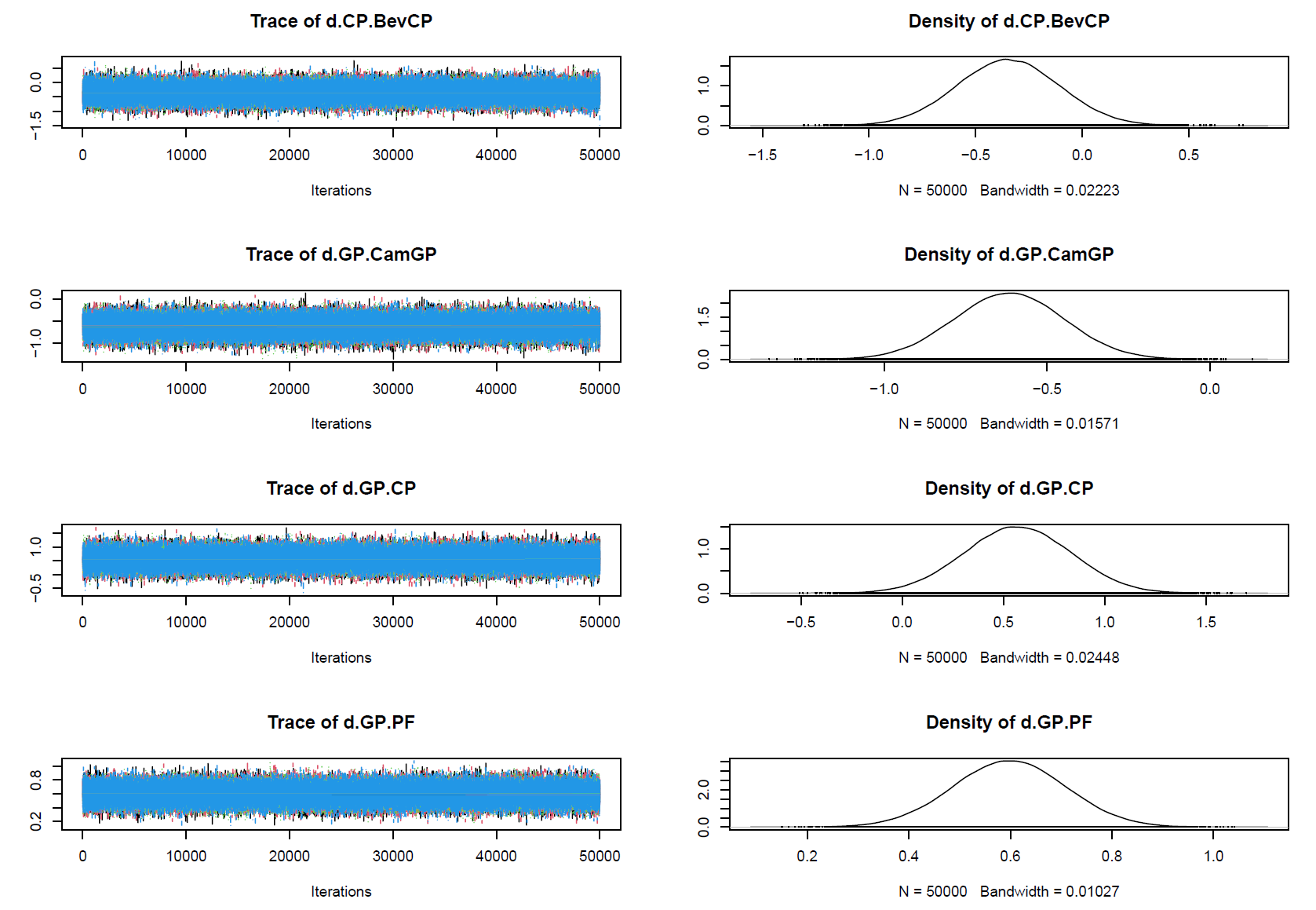


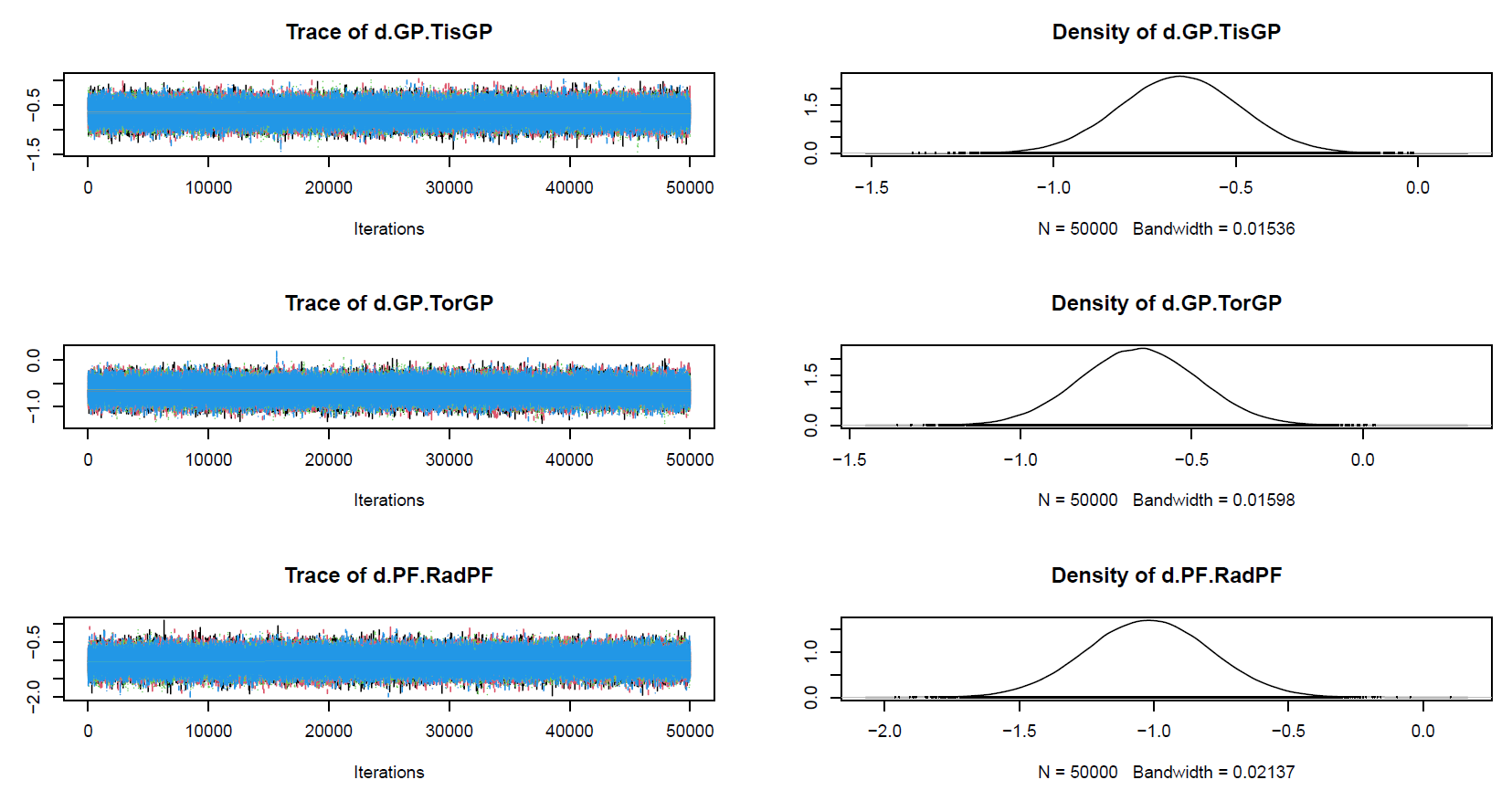


C History for objective response rate


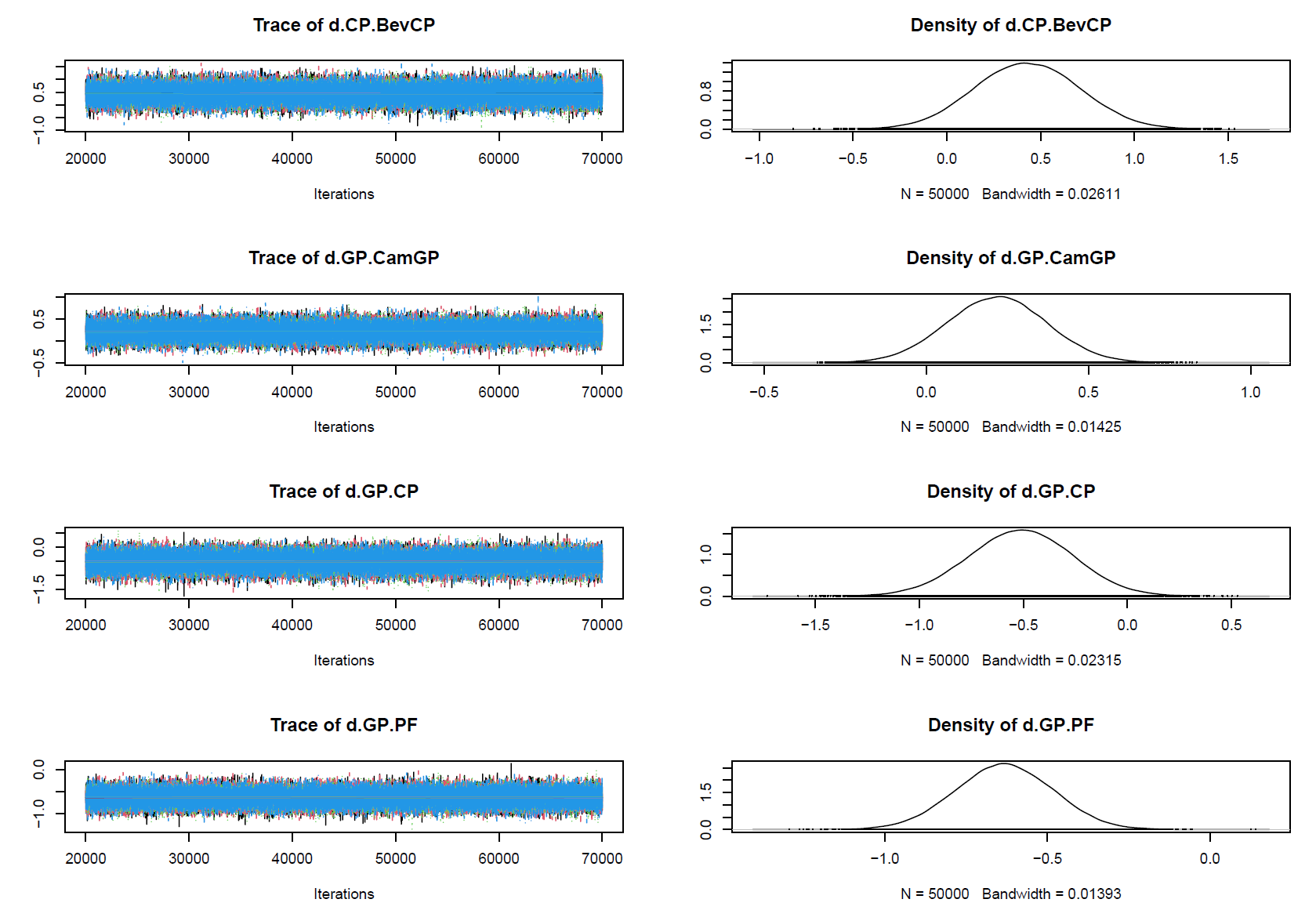


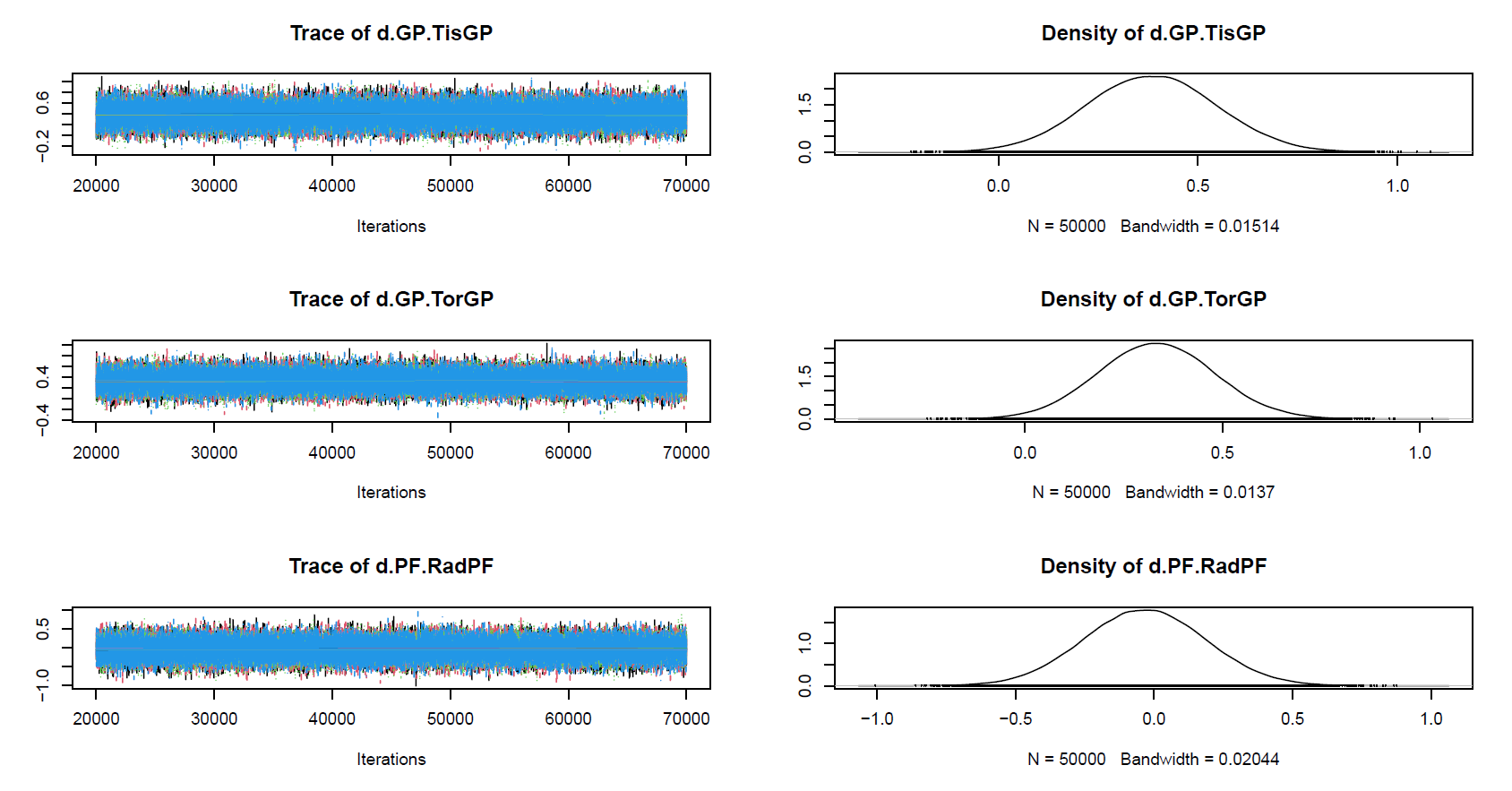


D History for grade ≥3 AEs


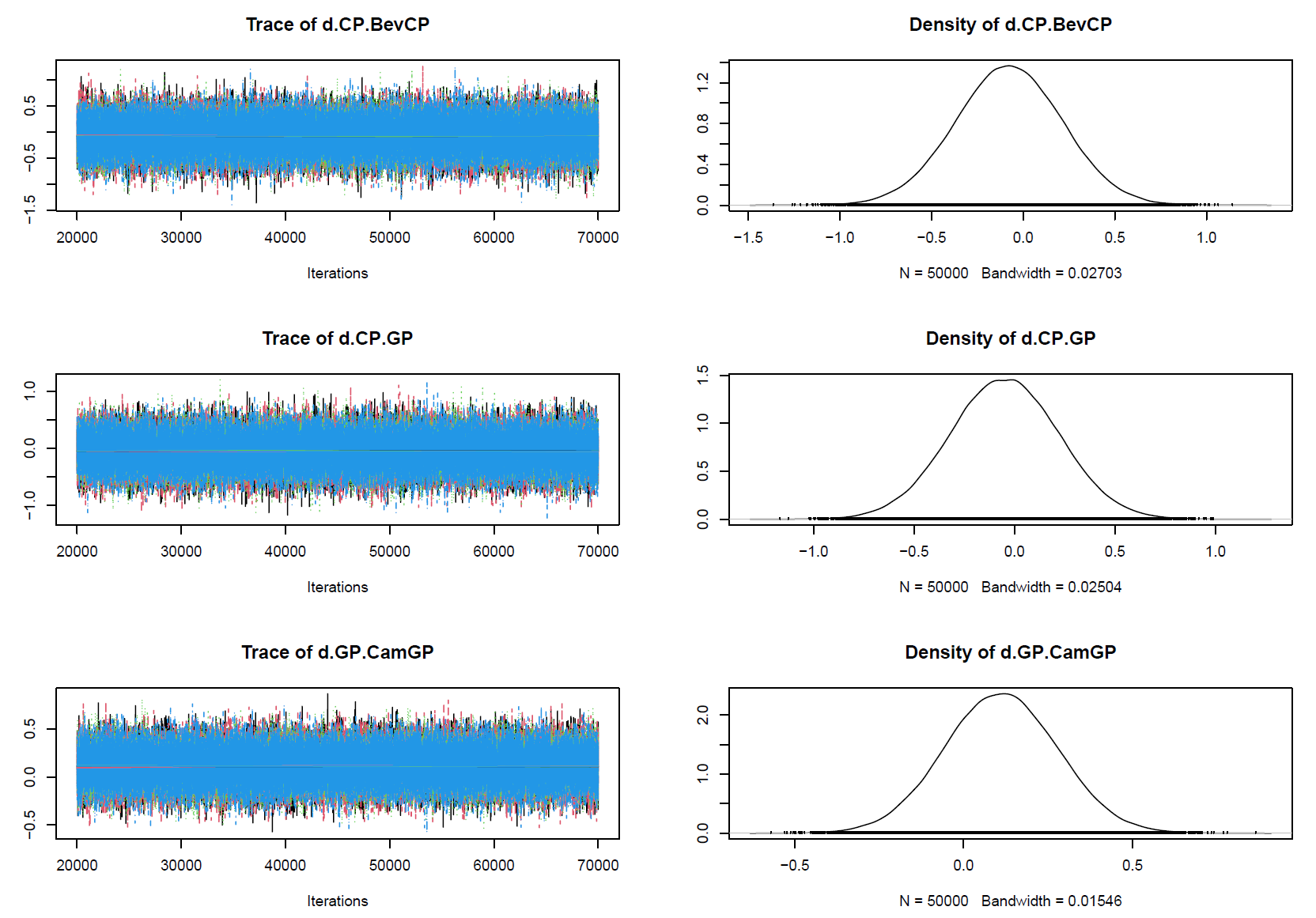


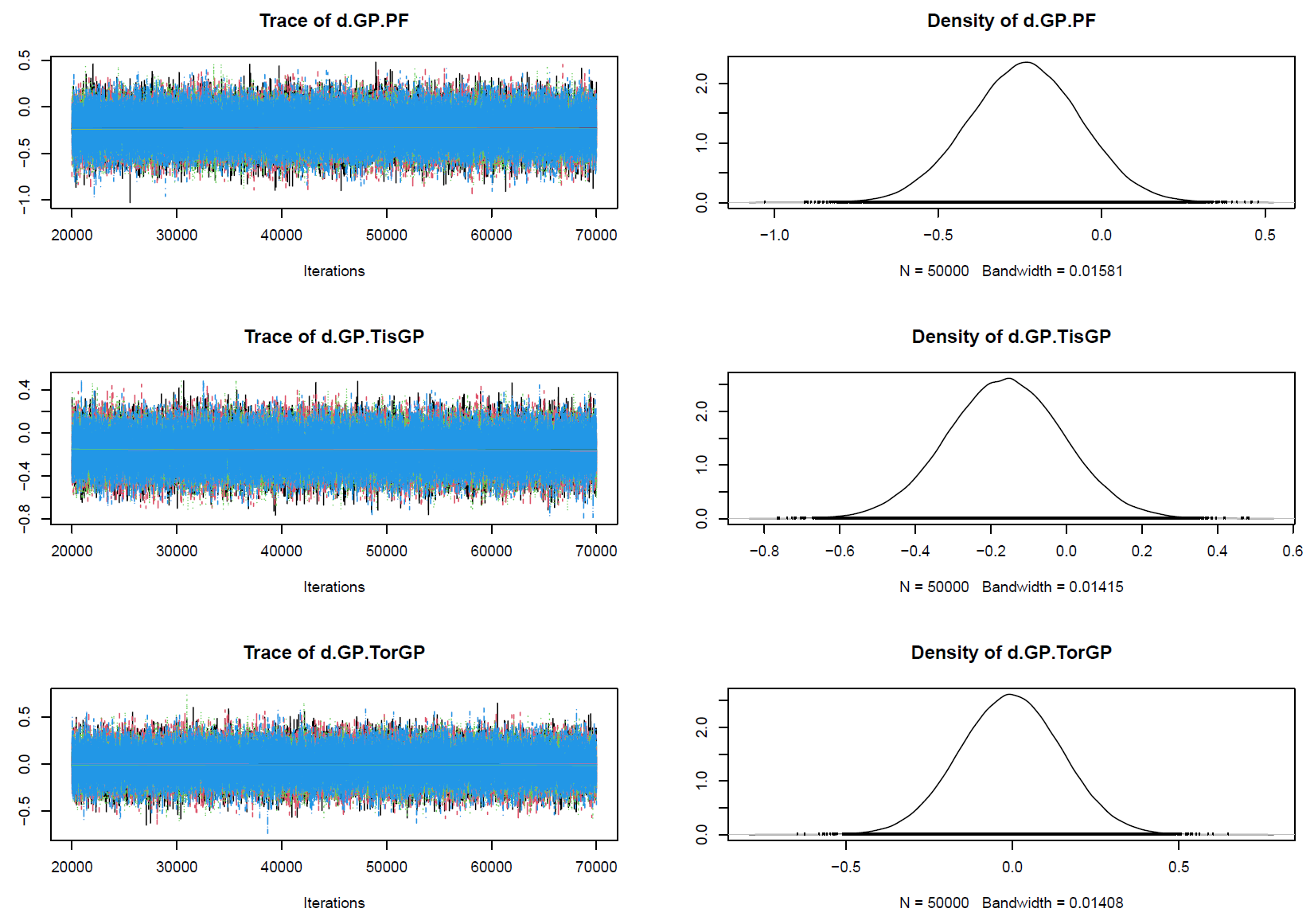


Figure 2. Convergence of the three Markov Chain Monte Carlo (MCMC) chains established by of the Brooks-Gelman-Rubin diagnostic for overall survival (A), progression-free survival (B), objective response rate (C), and grade ≥3 AEs (D).

A


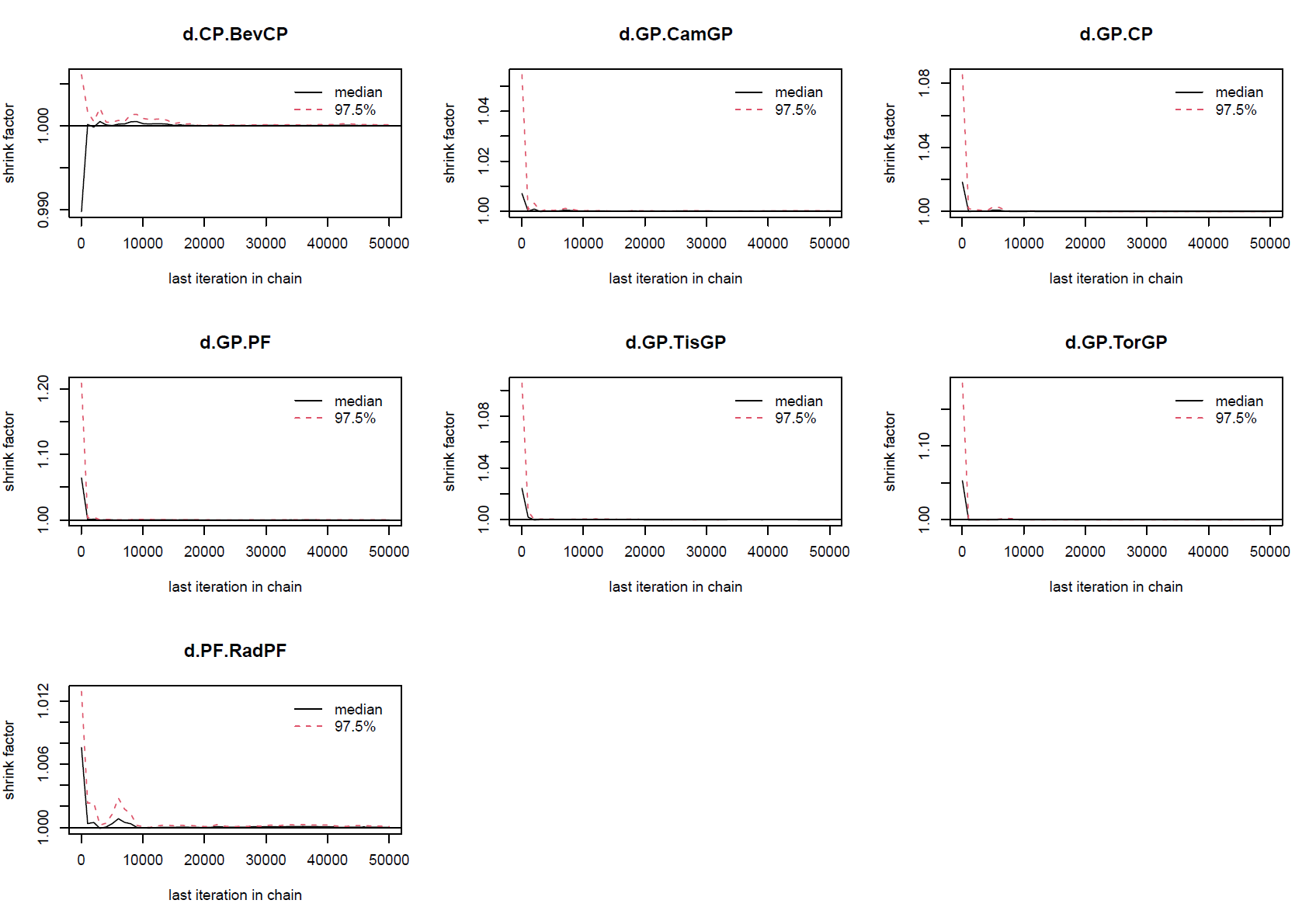


B


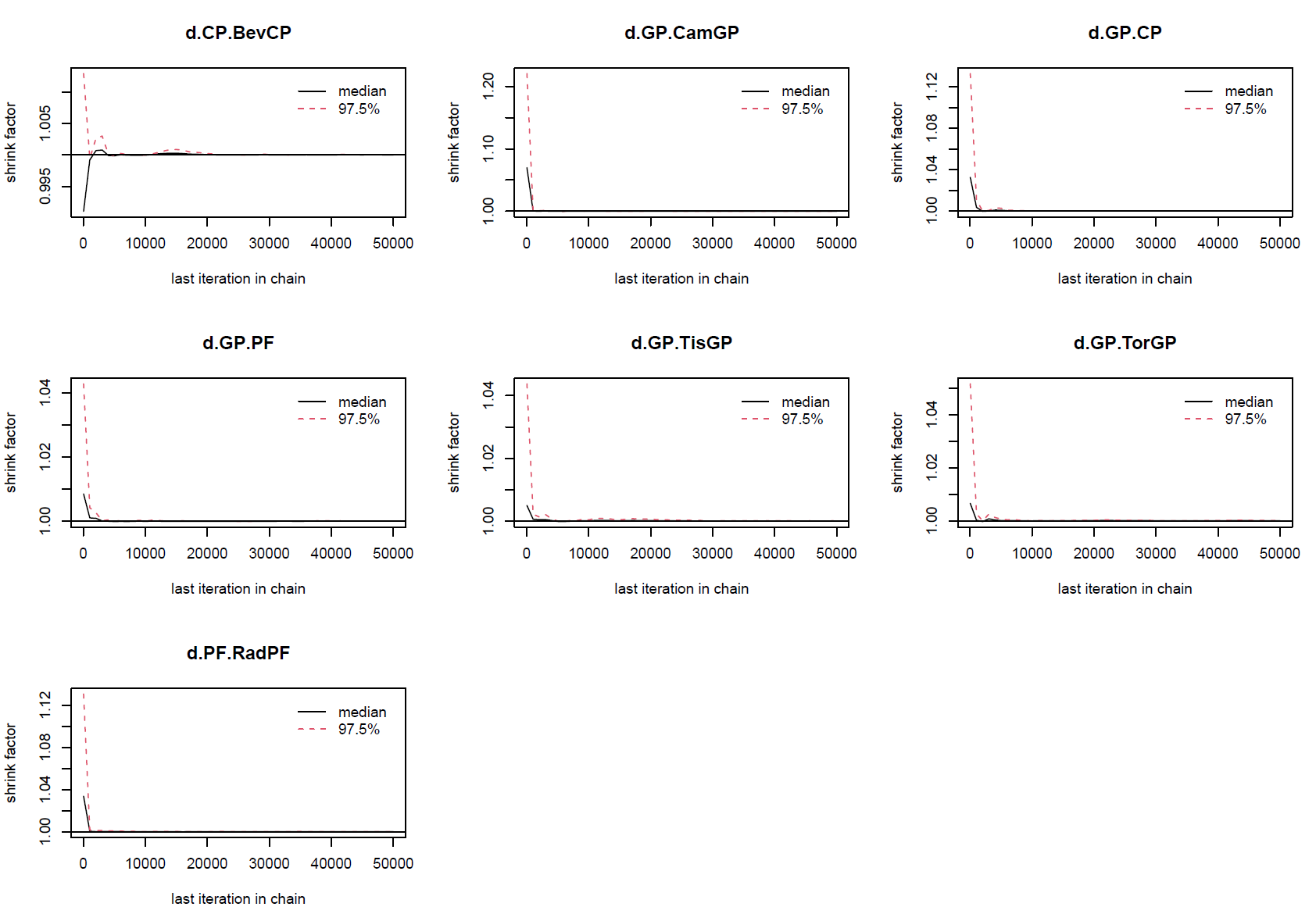


C


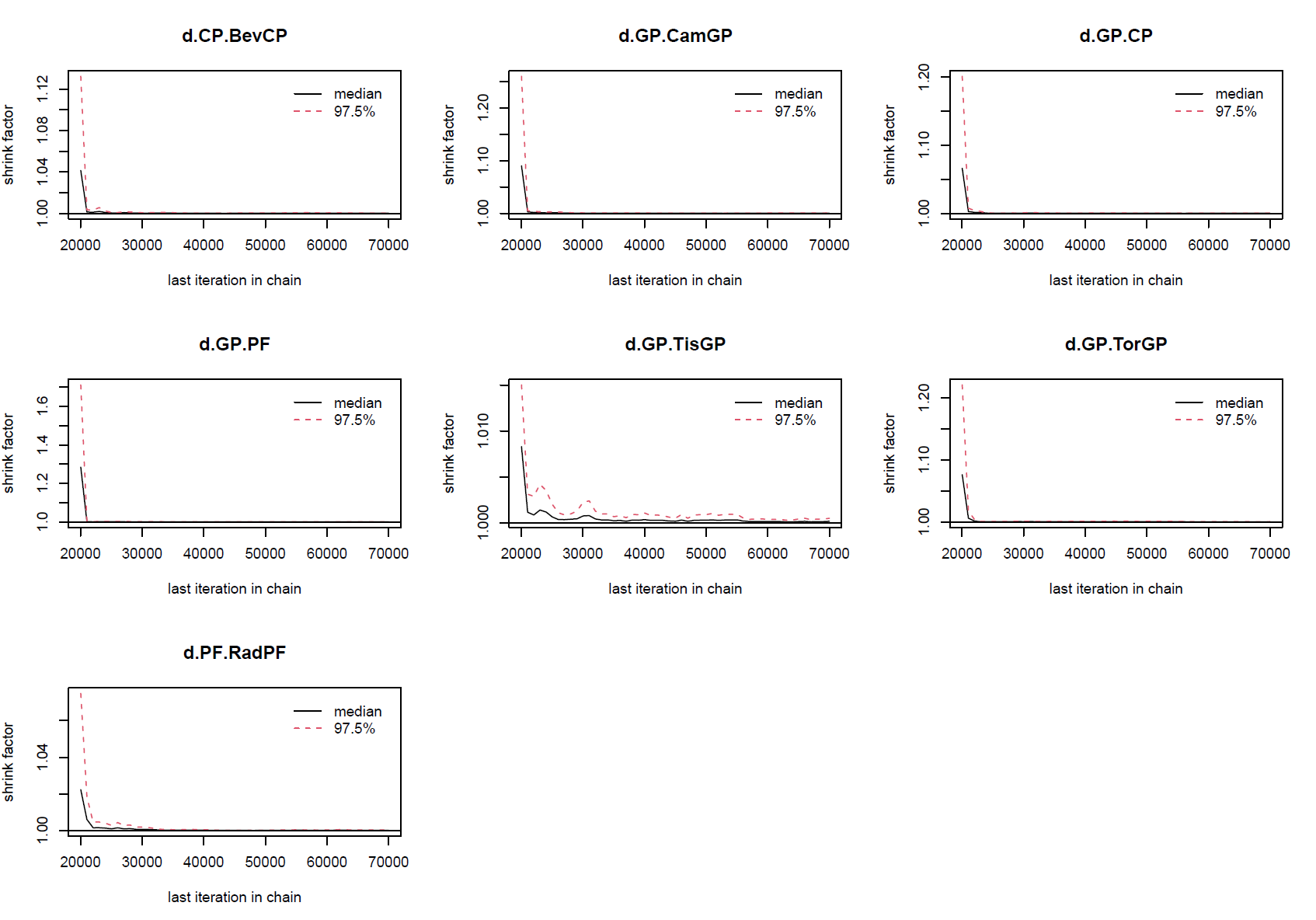


D


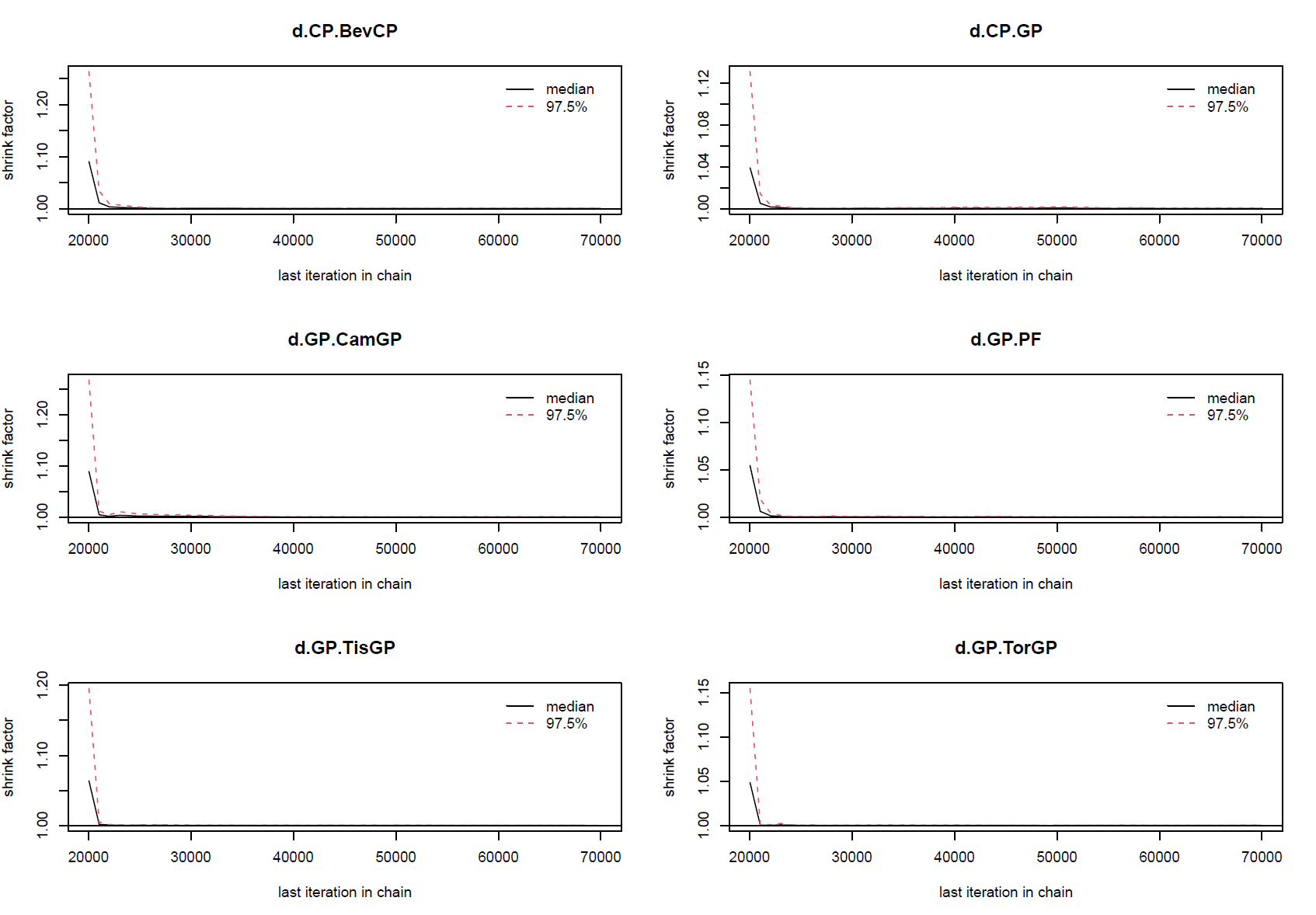

Supplement: Supplementary file 1 [file Table1.docx]
